# The Importance of Early Detection of Spinal Tumors Through Magnetic Resonance Imaging in Chiropractic Practices

**DOI:** 10.7759/cureus.51440

**Published:** 2024-01-01

**Authors:** Sharon Mok, Eric Chun-Pu Chu

**Affiliations:** 1 Chiropractic and Physiotherapy Clinic, New York Medical Group, Hong Kong, CHN

**Keywords:** chiropractor, chiropractic, magnetic resonance imaging, radiculopathy, back pain, spine tumor, schwannoma

## Abstract

Primary spinal tumors such as schwannomas are uncommon causes of back pain that can be easily missed during the initial workup. Delayed diagnosis is associated with further neurological impairment. A 46-year-old man presented with a six-month history of progressive lower back pain and left leg radiculopathy. Previous treatments failed, including medications, physical therapy, acupuncture, and chiropractic manipulations. Examination revealed weakness (4/5) in left knee extension and ankle dorsiflexion. Magnetic resonance imaging (MRI) revealed a 2-cm intraspinal schwannoma at the L4 level with nerve root compression. The patient underwent laminectomy and gross total resection without any complications. The patient had near-complete symptomatic resolution six weeks postoperatively and returned to normal functioning. After four months of postoperative rehabilitation, the patient remained asymptomatic. This case reinforces the urgent need for early MRI in the presence of neurological deficits and other symptoms, despite normal radiographs. An increased suspicion of spinal tumors can prevent delays in diagnosis and minimize adverse outcomes. Multidisciplinary care optimizes the treatment of complex cases.

## Introduction

Low back pain is a highly prevalent condition, affecting up to 80% of adults at some point in their lifetime [1]. Although often self-limiting and amenable to conservative management, low back pain can also indicate more serious underlying pathologies that require prompt diagnosis and referral. Primary spinal tumors are an important cause of back pain warranting timely identification. The annual incidence of primary intraspinal tumors is estimated at 1.5 per 100,000 individuals [2]. Schwannomas, originating from Schwann cells of the nerve sheath, are among the most common primary spinal tumors, with an incidence of 0.3-0.4 per 100,000 [3]. These slow-growing lesions are typically benign but can cause compression of neural elements, resulting in radicular pain, neurological deficits, and even spinal cord compression in some cases.

Owing to their rarity and symptom overlap with more common back pain etiologies, spinal schwannomas are often misdiagnosed and treated conservatively over a prolonged timeframe before a correct diagnosis is reached [4]. Chiropractors may utilize radiographic imaging as part of their assessment of spinal rehabilitation in Hong Kong. However, it is important to note that the routine use of imaging, including MRI, for uncomplicated low back pain is not recommended [5]. Reliance on clinical findings and plain radiography alone may be insufficient in detecting serious spinal pathologies in such cases. Delays in using advanced imaging modalities such as magnetic resonance imaging (MRI) can postpone the diagnosis of underlying tumors or other conditions that warrant prompt specialist referral. However, it is important to consider the current guidelines and flowchart for the appropriate use of MRI in the diagnostic process. These guidelines recommend reserving MRI for cases with specific indications, such as persistent or worsening symptoms, red flags, or neurological deficits, as outlined in the relevant clinical guidelines. In the context of these recommendations, the delay in obtaining an MRI in our presented case highlights the importance of timely and appropriate utilization of advanced imaging [6].

Herein, we present a case emphasizing the importance of utilizing MRI in chiropractic patients with specific clinical characteristics that justify the need for advanced imaging to screen for severe spinal pathologies. This report highlights the potential risks associated with relying solely on red flags and limited clinical and radiographic findings. Such reliance can lead to delayed diagnoses and the potential for adverse outcomes during the conservative treatment of undetected serious diseases.

## Case presentation

A 46-year-old man presented to our chiropractic clinic with a six-month history of left-sided lower back pain radiating to the posterior thigh. The pain had initially been dull and intermittent, but it progressed to a constant, sharp sensation over the last two months. Aggravating factors included lying down for over an hour, coughing, and morning toothbrushing, which elicited a sudden shooting pain in the left lower leg lasting 5-10 seconds. The pain disrupted sleep, allowing only three hours nightly. On the numerical rating scale, he rated the severity as 9. Side-lying provided mild relief, similar to extending the spine while lying down. Standing also alleviated the patient’s symptoms.

The patient’s medical history was unremarkable. The patient did not have constitutional symptoms, bowel/bladder changes, previous surgeries, recent injuries, or spinal injections. Social history was negative for smoking and notable only for social alcohol use. The family history did not include any cancer or spinal disorders. The baseline quality of life was 64% according to the World Health Organization’s Quality of Life assessment.

**Figure 1:**
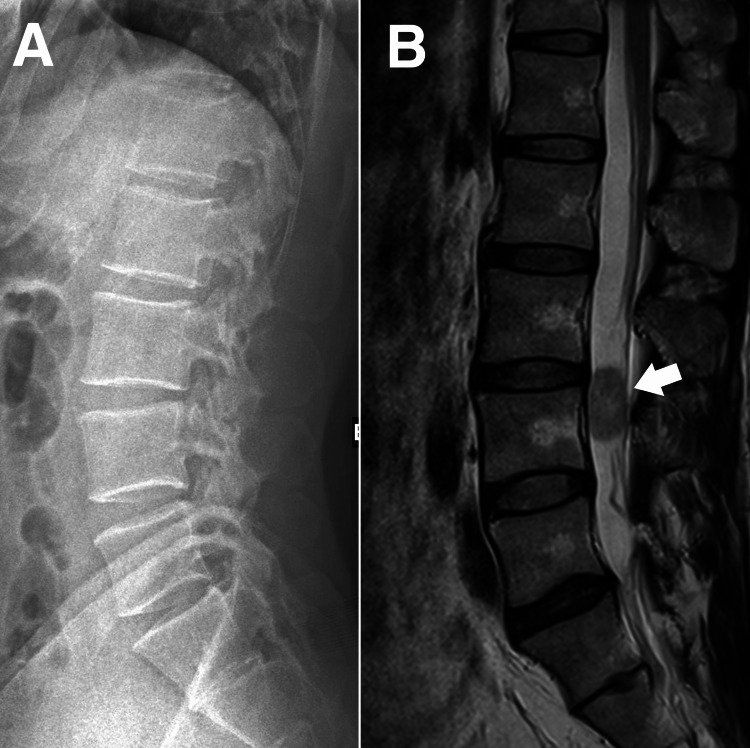
Radiological examinations of the lumbar spine. (A) Initial lumbar radiograph identified mild degenerative joint disease at the lumbar spine. (B) Six months after the initial onset, the chiropractor requested a magnetic resonance imaging of the lumbar spine, which revealed a suspicious oval T2 mild hypointense intrathecal mass at the L4 level, measuring 1.89 cm x 1.16 cm x 2.39 cm (white arrow). The nerve roots are compressed and displaced peripherally.

The patient first sought care six months before the onset of symptoms. Lumbar radiographs obtained by the primary care provider revealed multiple mild degenerative joint diseases (Figure 1A). The patient was diagnosed with lumbosacral radiculopathy and treated with a nonsteroidal anti-inflammatory drug (diclofenac) without improvement. Acupuncture and spinal manipulation by a traditional Chinese medicine practitioner also failed to provide relief.

Because of worsening pain over two months, the patient consulted a chiropractor for further care. A chiropractic evaluation revealed a normal lumbar range of motion, but end-range flexion increased left leg pain and tightness. Straight leg raise was negative on the right leg but exacerbated left leg tightness. Sacroiliac compression and prone extension tests showed increased left-sided lower back pain. Motor strength testing demonstrated weakness (4/5) in left knee extension and ankle dorsiflexion. The Hoffmann-Tinel sign was negative. Sensations and reflexes were intact.

Based on progressive neurological deficits, the chiropractor’s differential diagnosis included L5/S1 disc herniation with radiculopathy, lumbar spinal stenosis, piriformis syndrome, and facet joint syndrome. Because of worsening symptoms despite conservative care, the chiropractor requested an MRI. The MRI revealed a large intraspinal mass at L4 eccentric to the left, displacing the thecal sac and compressing the L4 nerve roots (Figure 1B). This was most consistent with a nerve sheath tumor such as a schwannoma. Owing to the size and neurological impact, the radiologist discussed the findings with the chiropractor and promptly referred the patient to an on-site spine surgeon who advised surgical resection. The patient underwent successful schwannoma resection without complications. At six-week follow-up, the patient showed near-complete resolution of symptoms and was able to return to normal activity levels. Postsurgical rehabilitation exercises and spinal mobilization therapy were recommended. At the fourth-month follow-up evaluation, his quality of life improved to 100%, and he remains asymptomatic.

## Discussion

Primary spinal tumors, although rare, should remain an important diagnostic consideration for primary healthcare practitioners when evaluating patients with back pain, especially when worrisome symptoms are present. They often manifest as radicular symptoms that mimic other causes of back pain, such as disc herniation or spondylosis [2]. In a recent retrospective analysis, radiculopathy was the most common presenting symptom (88%) among 50 cases of spinal schwannoma [3]. The average duration between symptom onset and definitive diagnosis is 16 months, which highlights a frequently insidious course [3]. In our case, the initial evaluation did not include a clear assessment for red flags to rule out serious pathologies, such as cancer, unexplained loss of vision, weight loss, immunosuppression, infection, intravenous drug use, or prolonged use of corticosteroids. This raises the importance of implementing red flag screening triage during the initial assessment of patients with low back pain. While the chiropractor's management at the six-month follow-up is not invalidated, it is essential to discuss the need for ongoing monitoring and clinical evaluation of patients with underlying serious pathology based on their profile.

Although patients with spinal tumors rarely present to chiropractic clinics [7], six cases of documented chiropractic management of patients who were later determined to have spinal schwannomas after delayed MRI have been reported [1,8-13]. Two previous cases were similar to our case, that is, with involvement of the lumbar spine and with radiculopathy [1,8]. Chu et al. described a similar case of a middle-aged man with lower back pain and leg symptoms originally diagnosed with discogenic radiculopathy who received conservative care, including chiropractic care before an MRI revealed a spinal tumor [1]. Caputo and Cusimano reported a case of a 37-year-old woman with a four-year history of progressive low back and leg pain and urinary and fecal incontinence and was ultimately diagnosed as having a T12-L2 spinal schwannoma [8]. Other types of tumors were also detected by MRI by chiropractic consultations [14-17].

Although chiropractic treatment may offer temporary relief of symptoms caused by spinal tumors, prompt advanced imaging is crucial when suspicious clinical symptoms such as neurological deficits are present [1]. Early diagnosis can prevent adverse outcomes of manipulative therapies and allow timely referral for surgical resection, which is the definitive treatment for these lesions. Benign schwannomas can cause spinal cord compression and permanent neurological damage if left untreated [4].

This case report highlights the importance of defining and justifying the threshold for MRI in the workup of suspected back pain presentations. To provide a more concrete and specific approach, we need to answer the question of which type of clinical presentations or patient profiles warrant the cost-effective utilization of MRI. It also indicates the need for specific recommendations about the screening of serious pathologies and their follow-up in chiropractic practice. It is important to consider the clinical profiles of patients with low back pain and provide targeted recommendations that take into account the known benign natural history of pain, where more than 90% of cases demonstrate a nonspecific course. These recommendations should prioritize cost-effectiveness and include strategies for early identification of red flags, appropriate utilization of diagnostic imaging modalities, multidisciplinary care coordination, and optimization of outcomes in chiropractic practice. As a single case report, this study has certain limitations, including the lack of standardized treatments, validated outcome measures, blinding [18], and extended follow-up. However, it is important to note that there is a grade of recommendation for exercise and education, which has been well-established in recent years.

## Conclusions

This case underscores the importance of maintaining a high index of suspicion for rare but serious causes of back pain such as spinal schwannomas when clinical symptoms are present, particularly worrisome neurological deficits refractory to conservative care. Early diagnosis allows prompt surgical resection before further cord or nerve damage. It is critical to discuss the clinical context and provide a clear justification for the timing of MRI indication, particularly at the initial presentation. This ensures that patients understand the specific circumstances in which an urgent MRI is necessary to prevent the risk of adverse outcomes, including permanent neurological damage. This discussion becomes especially important for patients who may have reservations or hesitations regarding MRI. Multidisciplinary management, through collaborative care among chiropractors, radiologists, and spine surgeons, is also key to providing integrated diagnosis and treatment. The discussion on diagnosis time in this report requires further elaboration to provide a deeper understanding of the factors influencing timely diagnosis, including specific criteria such as timeline and clinical findings. This report reinforces the essential role of MRI and integrated care models in the timely diagnosis and optimal treatment of suspected serious pathologies that cause back pain. In addition, it also provides a reminder to maintain vigilance and a low threshold for advanced imaging when clinical symptoms suggest the possibility of serious occult disease.
